# Characteristics and influencing factors of the first dental visit among children in Bangkok, Thailand: a cross-sectional study

**DOI:** 10.1186/s12903-023-03811-4

**Published:** 2024-01-03

**Authors:** Pornpailin Kasemkhun, Varangkanar Jirarattanasopha, Wannee Lertsooksawat

**Affiliations:** 1 Department of Pediatric Dentistry, Faculty of Dentistry, Mahidol University, Bangkok, Thailand; 2 Department of Pharmacology, Faculty of Dentistry, Mahidol University, Bangkok, Thailand

**Keywords:** Dental caries, First dental visit, Oral health

## Abstract

**Background:**

The first dental visit (FDV) is fundamental to good oral health. This study aimed to investigate the characteristics of FDV, including the influencing factors, in children attending the government dental hospital in Bangkok.

**Methods:**

This study included 289 pairs of new patients (aged 0–12 years) and their parents. The questionnaires, consisting of three parts: socio-demographic, reasons for the child’s FDV and for not having FDV earlier, and the children’s oral health knowledge and attitude, were completed by parents. The Chi-square and Kruskal-Wallis tests were used to compare variables among age groups. The level of statistical significance was set at *p* < 0.05. A multiple linear regression analysis was performed to identify the factors that influence the age at FDV. The variables included in the analysis were the child’s characteristics, the family’s socio-demographic background, the presence of symptoms, and the parents’ knowledge and attitude towards their child’s oral health.

**Results:**

The mean ages of children at FDV were 5.57 ± 2.88 (age range of 0.58–11.92) years. There were 2.42% who had FDV ≤ 12 months of age, and 76.5% of all children already had dental caries. A low attitude score among parents was significantly found in the older age group of children (*p* = 0.001). The influencing factors with the age at FDV were the parental age (*p* < 0.001), the presence of symptoms or chief complaints (*p* = 0.016), and the presence of dental caries (*p* < 0.001).

**Conclusions:**

Children in Bangkok had seriously delayed FDV compared to the recommended guidelines. The parental age strongly influenced the proper age at FDV, together with the presence of symptoms or chief complaints, and the presence of dental caries. Thus, an effective campaign to raise awareness about FDV is still needed, especially in advanced maternal age.

## Background

According to the recommendation of the American Academy of Pediatric Dentistry (AAPD) [1], American Dental Association (ADA) [2] and European Academy of Pediatric Dentistry (EAPD) [3], the first dental visit (FDV) is recommended at the time of the first tooth eruption and not later than 12 months of age. The objective is to provide parents with the anticipatory guidance and preventive counseling on dental health for children that can also establish the good attitudes in parents that will be passed on to their children and consequently improve the long-term quality of their lives. Moreover, it was found that the sooner a child is seen by a dentist, the smaller the number of treatment needs and the lower the cost of dental procedures [4].

Previous studies showed the different ages of a child’s first dental visit in different countries between the ages of 7 months and 14 years of age. Unfortunately, more than 50% show up with symptoms, and the most common reasons were pain and dental caries [5–10]. Although some of those came for only prophylactic reasons, the decayed teeth and poor oral hygiene were still detected. This demonstrates the misunderstanding of parents about their children’s oral health because the disease already existed in their child’s mouth. Thus, the anticipatory guidance in FDV should not be overlooked.

Numerous factors can possibly affect the time of the FDV, such as the socio-demographic characteristics, knowledge, and attitude of parents toward the oral health of themselves and their children. In developing countries, apart from socioeconomic status, it was found that the education level and attitude towards dental health of parents also played an important role in the child’s FDV due to their inverse relationship. In Thailand, the prevalence of dental caries was 52.9%, and the mean decayed teeth have already reached 2.8 per person in 3-year-old children, which could imply that the dental caries occurred instantly after the primary dentition had erupted completely [11].

Thus, characteristics of children’s FDV and the influencing factors affecting the proper period of the FDV are still needed for developing and providing an appropriate strategy to increase parents awareness of the FDV as it has never been specifically established before in Thailand. Therefore, this cross-sectional study is aimed at investigating the characteristics of FDV, including influencing factors, in children attending the government dental hospital in Bangkok, Thailand.

## Methods

### Ethical considerations and sample size

This cross-sectional study was conducted among children who were aged up to 12 years old and came for the FDV at the pediatric dental clinic of Mahidol University between October 2022 and March 2023, following the protocol that was approved by The Ethics Committee of Mahidol University, Thailand (MU-DT/PY-IRB 2022/DT115).

The sample size was determined by the total population size of the new patients, or 1,042 children who attended the pediatric dental clinic at the same time in the previous year, using Yamane’s formula [12] with 0.05 as the margin of error. The calculated sample size was 289. The inclusion criteria for this study were children aged up to 12 years old who came for their first dental visit and had never had a previous dental visit at any other dental clinic before. The exclusion criteria were a child whose parent could not give clear information about the child’s dental history.

### Data collection

The parents whose child met the inclusion criteria were selected by simple random sampling using random number method (two numbers each day) and gave their informed consent for themselves and their children, then completed the questionnaire which contained 3 parts: firstly, the socio-demographic characteristics: age and gender of their child, number of children in the family, education level of parent and monthly family income. Secondly, the reason for their child’s FDV and the reason for not having FDV earlier. Lastly, the knowledge and attitude of parents toward the oral health of their child (10 yes/no questions). The content validity was validated by two experienced pediatric dentists, and then the comprehension and clarity were tested with a group of parents as a pilot. The questions that confused the reader were revised and improved before use.

The children’s oral health status consisted of decayed teeth in both primary and permanent dentitions was examined by dental and pediatric resident students under the supervision of highly experienced pediatric dentists in that FDV as a normal situation without any interventions.

### Statistical analysis

The collected data were recorded in Microsoft Excel software. Then the data were analyzed using SPSS (version 23.0, IBM, Armonk, NY, USA). Age and reason for FDV, along with the socio-demographic characteristics of parents, were presented as descriptive statistics calculated and reported as frequencies, both minimum and maximum values, and the mean standard deviation. The chi-square test, Fisher’s exact test, and Kruskal-Wallis test were used to compare the differences between age groups regarding their baseline socio-demographic background, the reason for having FDV, the reason for not having FDV earlier, and their parents’ knowledge and attitude toward their child’s oral health. The Pearson’s correlation coefficient was calculated to assess the correlation between parental knowledge, parental attitude, and the age of FDV. The Spearman’s correlation coefficient was conducted to assess the correlation between all other variables in the study. The multiple linear regression analysis was conducted to determine which variables influenced the age of FDV. The level of statistical significance was set at 0.05.

## Results

A total of 289 pairs of parents and children were included in this study. The mean ages of children who attended the pediatric clinic for FDV were 5.57 ± 2.88, with a range of 0.58 to 11.92 years. Only 7 children (2.42%) had FDV not later than 12 months of age. The proportion of children’s genders was similar between girls and boys (46.4:53.6), and most children had siblings (63.3%). For parents, the majority of age range was 30–40 (51.9%), followed by 29.8% above 40. Almost three out of four were mothers (73.7%) who took responsibility for the child’s FDV, followed by fathers and grandparents. About 78.2% of parents graduated at least with a bachelor’s degree. The monthly family income was less than the average amount in Bangkok (30,000 THB) for 44.6%, as shown in Table 1.

**Table 1:** Socio-demographic characteristics

Characteristics	n		(%)
**Children Age**			
0–1	7		(2.4)
> 1–3	64		(22.2)
> 3–6	89		(30.8)
> 6–12	129		(44.6)
**Children’s gender**			
Girl	134		(46.4)
Boy	155		(53.6)
**Having siblings**			
Yes	183		(63.3)
No	106		(36.7)
**Parental Age**			
< 20	20		(6.9)
20–29	33		(11.4)
30–40	150		(51.9)
> 40	86		(29.8)
**Parent’s education level**			
Under 12th Grade or equal	63		(21.8)
Bachelor’s degree	166		(57.4)
Above or equal to Master’s degree	60		(20.8)
**Monthly family income (THB)**			
≤ 20,000	71		(24.6)
20,001–30,000	58		(20.0)
> 30,001	160		(55.4)
**Reasons for having FDV**			
Dental check-up	105		(36.3)
Dental caries	99		(34.3)
Pain	41		(14.2)
Trauma	7		(2.4)
Malocclusion	24		(8.3)
Others	13		(4.5)
**Reasons for not having FDV earlier**			
No symptoms	174		(60.2)
No time	51		(17.6)
Cooperative problem	16		(5.5)
Financial problem	47		(16.3)
Others	1		(0.4)

For oral health status, the mean decayed teeth in children aged 0–3 and > 3–6 were 3.65 ± 5.38, 7.70 ± 6.59 respectively. For primary and permanent dentition in group > 6–12, it was 5.02 ± 4.14 and 1.38 ± 2.12 respectively. There were 68 caries-free children (23.5%). For knowledge and attitude questions, the mean scores were 3.54 ± 1.04 and 3.23 ± 1.41 respectively. Focusing on the FDV-related knowledge question, it was found that 170 parents or 58.8%, answered correctly that they should bring their child for FDV since the first tooth has erupted.

**Fig. 1:**
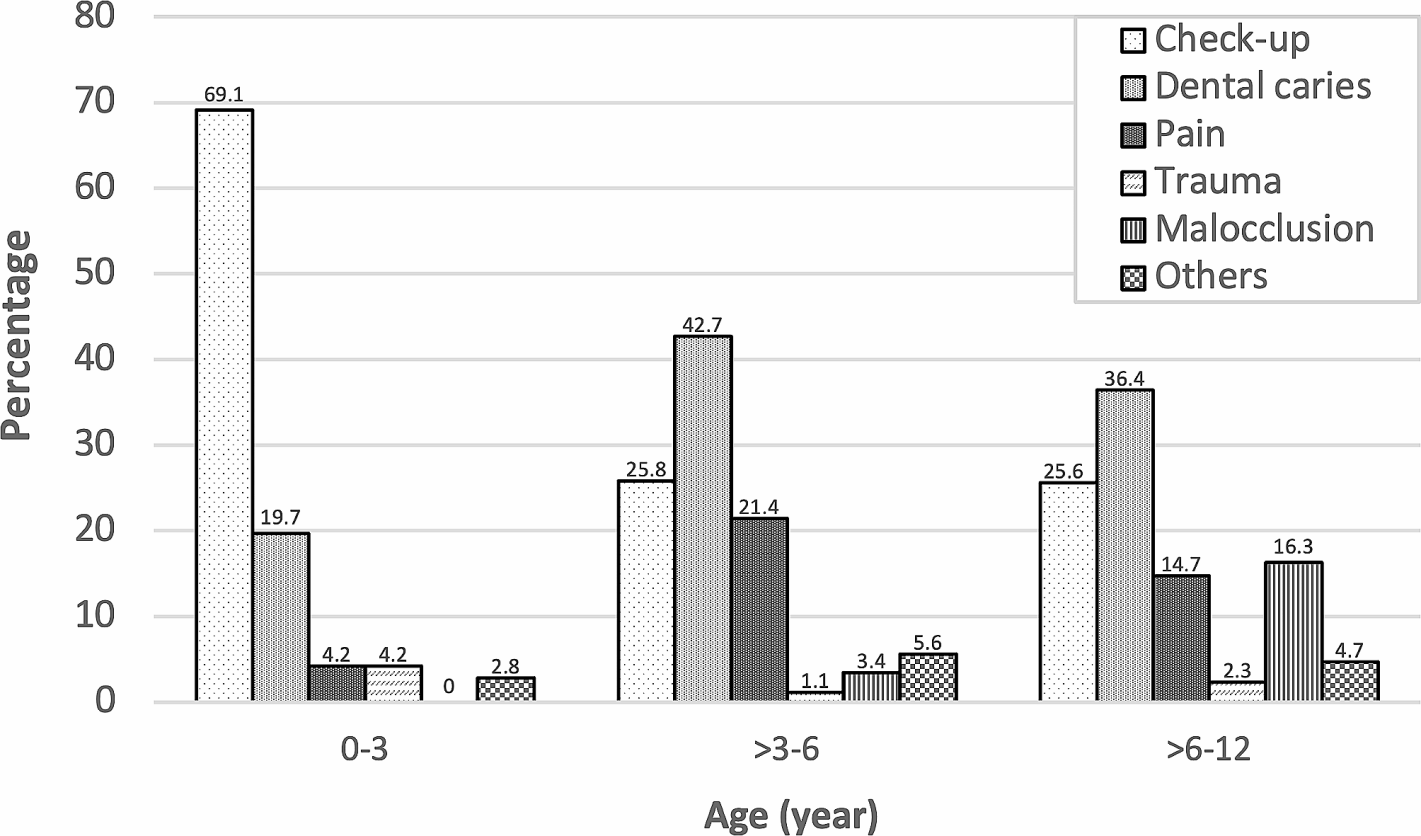
Percentage distribution of reasons for the first dental visit in each age group

The reason for FDV was mostly the dental check-up without symptomatic reasons, followed by the symptomatic reasons or chief complaints, which were dental caries, pain, malocclusion, trauma, and others (tooth mobility, swelling, abscess, bruxism, missing and retained primary teeth). While the most common reason for not having FDV earlier was that children showed no symptoms or chief complaints about their oral health, one parent claimed that her child’s FDV was postponed according to the Covid-19 pandemic. However, when categorized into age groups as Fig. 1, it was found that younger children had a distinct number of dental check-ups without symptomatic reasons, which was contrary to the older age group. Malocclusion was notably found in the > 6–12 group. Focusing on all children who came for a dental check-up without symptomatic reason, caries had already existed for 54.3%.

Table 2 compares the differences between age groups regarding their socio-demographic background, the cause of having FDV, the reason for not having FDV earlier, and their parents’ knowledge and attitude regarding their child’s oral health. The analysis revealed that parental age (*p* < 0.001), the presence of siblings (*p* = 0.038), the presence of FDV-related symptoms or chief complaints (*p* < 0.001), the presence of dental caries (*p* = 0.001), and parental attitude (*p* = 0.001) differed significantly among age groups. Moreover, the age at FDV exhibited a statistically significant positive correlation with parental age (*p* < 0.001), the presence of siblings (*p* = 0.049), and the presence of FDV-related symptoms or chief complaints (*p* < 0.001). Furthermore, it demonstrated a statistically significant negative correlation with parental attitude (*p* = 0.004), as presented in Table 3. It could be interpreted that the oldest age group tended to have higher parental age, have siblings, present with symptoms or chief complaints, have dental caries, and have lower parents’ attitude scores. However, only parental age (*p* < 0.001) and parental attitude (*p* = 0.004) showed a significant correlation with age at FDV. The result of multiple linear regression analysis demonstrated that the parental age (ß=0.951; *p* < 0.001), the presence of symptoms or chief complaints for FDV reasons (ß=0.831; *p* = 0.016), and the presence of dental caries (ß=2.080; *p* < 0.001) were the potential factors influencing the age of FDV, as presented in Table 4.

**Table 2:** Differences of characteristics among age groups

Characteristics	Age group n(%)			*p*-value
	0–3	> 3–6	> 6–12	
**Gender**				0.496
Girl	36(50.7)	37(41.6)	61(47.3)	
Boy	35(49.3)	52(58.4)	68(52.7)	
**Parental Age**				< 0.001*
< 20	6(8.5)	8(9.0)	6(4.7)	
20–29	8(11.3)	13(14.6)	12(9.3)	
30–40	43(60.5)	55(61.8)	52(40.3)	
> 40	14(19.7)	13(14.6)	59(45.7)	
**Having siblings**				0.038*
Yes	36(50.7)	59(66.3)	88(68.2)	
No	35(49.3)	30(33.7)	41(31.8)	
**Parent’s education level**				0.064
Under 12th Grade or equal	9(12.7)	21(23.6)	33(25.6)	
Bachelor’s degree	42(59.2)	56(62.9)	68(52.7)	
Above or equal to Master’s degree	20(28.2)	12(13.5)	28(21.7)	
**Monthly family income (THB)**				0.316
≤ 20,000	12(16.9)	26(29.2)	33(25.6)	
20,001–30,000	13(18.3)	16(18.0)	29(22.5)	
> 30,001	46(64.8)	47(52.8)	67(51.9)	
**Reasons for having FDV**				< 0.001*
Presence of symptoms/chief complaints (dental caries, pain, trauma, malocclusion, others)	22(31.0)	66(74.2)	96(74.4)	
No symptoms/complaints (dental check-up)	49(69.0)	23(25.8)	33(25.6)	
**Reasons for not having FDV earlier** ^**a**^				0.001*
No symptoms	38(53.5)	57(64.0)	49(61.2)	
No time	11(15.5)	10(11.2)	30(23.3)	
Cooperative problem	1(1.4)	5(5.6)	10(7.8)	
Financial problem	21(29.6)	16(18.0)	10(7.8)	
Others	0(0)	1(1.2)	0(0)	
**Presence of dental caries**				< 0.001*
Yes	34(47.9)	73(82.0)	114(88.4)	
No	37(52.1)	16(18.0)	15(11.6)	
	**(Mean ± SD)**			
**Knowledge score** ^**b**^	3.79 ± 0.98	3.42 ± 1.15	3.49 ± 0.99	0.085
**Attitude score** ^**b**^	3.41 ± 1.30	3.21 ± 1.39	2.94 ± 1.40	0.001*

^a, b^ Data was analyzed by Fisher’s exact test and Kruskal-Wallis test respectively, whereas other socio-demographic characteristics and oral status were analyzed by the Chi-square test

*Statistically significant difference at *p*-value < 0.05

**Table 3:** Correlation coefficients between study variables

	Child’s gender	Having siblings	Parental age	Parental’s education level	Monthly family income	Presence of symptoms/chief complaints	Presence of dental caries	parent’s oral health knowledge score	parent’s oral health attitude score	Age at first dental visit
Child’s gender	1.00									
Having siblings	0.070	1.00								
Parental age	0.104	0.078	1.00							
Parental’s education level	− 0.051	− 0.042	0.273**	1.00						
Monthly family income	0.067	− 0.077	0.245**	469**	1.00					
Presence of symptoms/chief complaints	− 0.024	0.094	− 0.051	− 0.124*	− 0.163**	1.00				
Presence of dental caries	0.024	0.195**	− 0.040	− 0.160**	− 0.258**	0.401**	1.00			
Parent’s oral health knowledge score	− 0.026	− 0.094	-0.038	0.246**	0.212**	− 0.017	-0.099	1.00		
Parent’s oral health attitude score	0.030	− 0.007	0.053	0.255**	0.251**	− 0.126*	− 0.186**	0.391**	1.00	
Age at first dental visit	0.009	0.116*	0.253**	− 0.093	− 0.039	0.279**	0.374**	− 0.093	− 0.388**	1.00
** *p*-value < 0.01, * *p*-value < 0.05										

**Table 4:** Multiple linear regression analysis of age at first dental visit

Affecting factors	ß	95% CI	*p*-value
Parental age	0.951	(0.58,1.32)	< 0.001*
Presence of symptoms/chief complaints	0.831	(0.16,1.50)	0.016*
Presence of dental caries	2.080	(1.31,2.85)	< 0.001*

Note: R = 0.467 and R^2^ = 0.218. Excluded non-significant variables: child’s gender, having siblings, patent’s education level, monthly family income, parent’s oral health knowledge and attitude score

*Statistically significant difference at *p*-value < 0.05

## Discussion

The first dental visit should take place at the time of the first tooth eruption and not later than 12 months of age, as stated by the widely used worldwide pediatric dentistry guidelines [1–3]. This seems like a long way to go for children in Bangkok. According to the present study, the mean ages of FDV were 5.57 ± 2.88 years, which was about four times later than the recommended age, and only 2.42% that straightly followed the recommendation. However, this result was in the midst of other studies from various countries: Poland (3.79 ± 1.82) [7], Turkey (3.64 ± 1.32) [13] and Bulgaria (51.9% at range of 3–6 years of age) [15], Nigeria (7.9 ± 3.7) [9], India (57% at range 6–9 years of age) [5] and Nepal (52.7% at range 7–11 years of age) [14]. The reason for the quite low number from the first two countries could be that the first study was conducted in a private dental clinic, not a government hospital like other studies and the following one included only preschool children, which both referred to a different group of samples from the current study. In keeping with the mean ages at FDV, the percentage of children who had FDV not later than 12 months of age was not much different when compared to Nigeria (0.8%) [9] and Turkey (2.9%) [13] and Bulgaria(1.73%) [15]. Nonetheless, these numbers were far less than the study performed in Brazil (10.1%) which focused only on the infants’ group in urban areas with a ten times larger number of samples [16]. Furthermore, the oral health status at FDV in our study was comparable to the earlier study in a developed country with good public dental services like Poland: 5.02 ± 4.14 vs. 4.50 ± 3.80 and 1.38 ± 2.12 vs. 0.50 ± 1.00 in primary and permanent dentition, respectively [17]. The presence of dental caries was noticeably associated with late FDV. It could imply that the earlier the children come, the better oral health they can have, as FDV is able to establish good oral hygiene due to the proper anticipatory guidance and disease management that will be provided in time by the dental practitioners.

The most common reason for FDV was a dental check-up without any symptoms or chief complaints, which corresponded to earlier studies [13, 17, 18]. Meanwhile, the reason for dental pain (14.2%) was less than half when compared with Mika et al. [7] (60.0%), Meera et al. [8] (42.04%), Olatosi et al. [9] (33.1%), and Ghimire et al. [14] (32.4%). This signified that most of the parents in this study still have concern for their child’s oral health and would not wait until the child had pain, even though it was quite late compared to the recommended age. In addition, it was obvious that having no symptoms did not mean there was no dental caries, as 54.3% of children came for a dental check-up without knowing that caries had already occurred. According to age groups, the youngest age group mostly came only for a check-up without symptoms, while caries could be detected more in older age groups. This could infer that caries might turn into a late stage as parents can detect it themselves, along with symptoms in some cases. Thus, if children had early FDV regardless of the caries presence, the severity would be less and the proper treatments could be provided in time, which would also be able to establish a good attitude in children rather than waiting until the symptoms showed up and negatively affected their behavior, which was in agreement with Rantavuori et al. [19] and Lin et al. [20]. Likewise, malocclusions that were mostly found in children in oldest age group with early mixed dentition, which is normal, would decrease in number if the child had an earlier FDV and a regular dental check-up.

Considering the socio-demographic factors that influence the proper age for FDV, it was found that parental age was a strong predictor, as a tendency toward late FDV was detected in parents with older ages, which was supported by the previous study [21]. Children who did not have siblings tended to have FDV at earlier ages compared to children with siblings. This could be due to the fact that attention and carefulness would be gained more from only-child parents and would not be shared by other children in the family. In addition, it was unsurprising that only 58.8% knew that they should bring their child for FDV since the first tooth has erupted, and it was also lower among parents in Puducherry, India (25%) [5], and Riyadh, Saudi Arabia (15.5%) [6]. This result confirms that the guidelines for FDV have not properly reached parents throughout the world. In contrast, parents with higher oral health attitude scores seemed to take their children earlier for FDV. Thus, providing and reinforcing a child’s good oral health attitude to parents as early as possible with continuity since the pregnancy period, especially in advanced maternal age, could potentially help them reach the goal of the proper age for FDV as recommended by the guidelines.

Moreover, it was interesting that there was a huge misunderstanding that having no symptoms of the child meant it was normal to not have FDV, as it was the most common reason for deferring the FDV, not a barrier of socioeconomic status like a financial or time problem. Some parents thought that the child’s noncooperation was one of the FDV barriers, which agreed with parents in Saudi Arabia [6]. This result indicated that it was necessary that the oral health practitioners inform and assure the parents about the level of cooperation related to the age of the children, as it is usual for children to be uncooperative in the early years of live.

The limitation of the present study was that different recorders might influence the obtainable data. Moreover, even though the pediatric dental clinic of Mahidol University included the most diverse socio-demographics of patients and parents throughout Bangkok, the fact that this was the only setting might limit the findings of this study, and some factors should have been considered, such as health insurance benefits. Future studies should focus on an effective campaign to promote an early first dental visit in the prenatal period, especially in advanced maternal age.

## Conclusion

Based on the findings of this cross-sectional study, it can be concluded that children in Bangkok had seriously delayed FDV compared to the recommended guidelines (≤ 12 months of age), together with an insufficiency of knowledge regarding FDV among parents. The most common reason for FDV was a dental check-up, with less than half having caries-free teeth. The influencing factors affecting the proper period of FDV were the parental age, the presence of symptoms or complaints, and the presence of dental caries. Therefore, the correct information regarding age, essentials, and benefits of FDV is needed and should be provided ahead of time in pursuance of bringing their child earlier to the proper period of FDV, as stated by recommendations to provide parents with anticipatory guidance and preventive counseling on dental health for children as its primary objective.

## Data Availability

The data that support the findings of this study are available from the corresponding author upon reasonable request.

## Abbreviations

ß: Beta coefficients
CI: Confidence interval
FDV: First dental visit
R: Multiple correlation coefficient
R^2^: Coefficient of determination
SD: Standard deviation
THB: Thai Baht
