# Correction: Characteristics and influencing factors of the first dental visit among children in Bangkok, Thailand: a cross-sectional study

**DOI:** 10.1186/s12903-026-08979-z

**Published:** 2026-06-30

**Authors:** Pornpailin Kasemkhun, Varangkanar Jirarattanasopha, Wannee Lertsooksawat

**Affiliations:** 1 Department of Pediatric Dentistry, Faculty of Dentistry, Mahidol University, Bangkok, Thailand; 2 Department of Pharmacology, Faculty of Dentistry, Mahidol University, Bangkok, Thailand

**Correction: BMC Oral Health 24, 11 (2024)**

**https://doi.org/10.1186/s12903-023-03811-4**

In the Ethical Considerations and Sample Size subsection of the Methods section and the **Ethical approval and consent to participant** section of this article [1], the author inadvertently provided the project number MU-DT/PY-IRB 2022/DT115 instead of the official ethics approval certificate number MU-DT/PY-IRB 2022/050.2909.
